# Low prevalence of *Plasmodium* and absence of malaria transmission in Conakry, Guinea: prospects for elimination

**DOI:** 10.1186/s12936-016-1230-9

**Published:** 2016-03-18

**Authors:** Bernard L. Kouassi, Dziedzom K. de Souza, Andre Goepogui, Siradiou M. Balde, Lamia Diakité, Arsène Sagno, Georgina I. Djameh, Frédérique Chammartin, Penelope Vounatsou, Moses J. Bockarie, Jürg Utzinger, Benjamin G. Koudou

**Affiliations:** UFR Science de la Nature, Université Alassane Ouattara, 02 BP 801, Abidjan, 01 Côte d’Ivoire; Centre Suisse de Recherches Scientifiques en Côte d’Ivoire, 01 BP 1303, Abidjan, 01 Côte d’Ivoire; Swiss Tropical and Public Health Institute, P.O. Box, CH-4002, Basel, Switzerland; University of Basel, P.O. Box, CH-4003, Basel, Switzerland; Noguchi Memorial Institute for Medical Research, P.O. Box LG 581, Legon-Accra, Ghana; Programmes National de Lutte contre l’Onchocercoses et les autres Maladies Tropicales Négligées, Ministère de la Sante, Conakry, Guinea; Filariasis Programme Support Unit, Liverpool School of Tropical Medicine, Pembroke Place, Liverpool, L3 5QA UK

**Keywords:** Malaria, Transmission, Bio-ecology, *Anopheles*, *Plasmodium*, Guinea

## Abstract

**Background:**

Over the past 15 years, mortality and morbidity due to malaria have been reduced substantially in sub-Saharan Africa and local elimination has been achieved in some settings. This study addresses the bio-ecology of larval and adult stages of malaria vectors, *Plasmodium* infection in *Anopheles gambiae s.l.* in the city of Conakry, Guinea, and discusses the prospect for malaria elimination.

**Methods:**

Water bodies were prospected to identify potential mosquito breeding sites for 6 days each in the dry season (January 2013) and in the rainy season (August 2013), using the dipping method. Adult mosquitoes were collected in 15 communities in the five districts of Conakry using exit traps and indoor spraying catches over a 1-year period (November 2012 to October 2013). Molecular approaches were employed for identification of *Anopheles* species, including *An. coluzzii* and *An.gambiae s.s*. Individual *An.gambiae* mosquitoes were tested for *Plasmodium falciparum* and *P. vivax* sporozoites using the VecTest™ malaria panel assay and an enzyme-linked immunosorbent assay. A systematic research of Ministry of Health statistical yearbooks was performed to determine malaria prevalence in children below the age of 5 years.

**Results:**

*Culex* larval breeding sites were observed in large numbers throughout Conakry in both seasons. While *Anopheles* larval breeding sites were less frequent than *Culex* breeding sites, there was a high odds of finding *An.gambiae* mosquito larvae in agricultural sites during the rainy season. Over the 1-year study period, a total of 14,334 adult mosquitoes were collected; 14,135 *Culex* (98.6 %) and 161 (1.1 %) from the *An.gambiae* complex. One-hundred and twelve *Anopheles* mosquitoes, mainly collected from rice fields and gardens, were subjected to molecular analysis. Most of the mosquitoes were *An*. *gambiae s.s.* (n = 102; 91.1 %) while the remaining 10 (8.9 %) were *An.melas.* The molecular M form of *An*. *gambiae s.s*. was predominant (n = 89; 79.5 %). The proportions of *kdr* genotype in the *An*. *gambiae s.s.* M and S form were 65.2 and 81.8 % (n = 9), respectively. No sporozoite infection were detected in any of the mosquitoes tested. The prevalence of *Plasmodium* recorded in children aged below 5 years was relatively low and varied between 2.2 and 7.6 % from 2009 to 2012.

**Conclusions:**

The low density of larval and adult stages of *Anopheles* mosquitoes, the absence of infected *An.gambiae* species and the low prevalence of *Plasmodium* in under 5-year-old children are important features that might facilitate malaria elimination in Conakry. The heterogeneity in species composition and resistance profiles call for vector control interventions that are tailored to the local bio-ecological setting.

## Background

Malaria is one of the most important diseases of poverty and its public health relevance, particularly in sub-Saharan Africa, cannot be overemphasized [1]. Yet, over the past 15 years, significant progress has been made, as malaria prevalence has been reduced by 50 %, as shown by Blatt and colleagues [2]. Prevention, particularly through insecticide-treated net (ITN) distribution and other vector control measures, was key in cutting down malaria transmission, clinical episodes and mortality. However, the effectiveness of vector control interventions depends on accurate information regarding distribution and abundance of the main vector species and current levels of insecticide resistance [3]. There is a critical need for a better understanding of the ecology of malaria vectors for control programmes to succeed. Study of spatial and temporal changes in anopheline mosquito abundance, quantification of transmission potential of vector populations, and description of distributions of host populations [4] are necessary prerequisites for predicting high-risk areas and implementing an effective disease control programme [5].

In Guinea, malaria is the leading cause of morbidity, hospitalization, clinical consultations in regional paediatric services [6, 7]. Moroever, according to the Global Burden of Disease (GBD) report in 2010, malaria is the leading cause of death in the population accounting for 22.5 % of all causes of death [8]. Malaria is endemic throughout the country, with holo-endemicity in Lower Maritime Guinea where the capital, Conakry, is situated [9]. From the first reports of Laveran in 1904 to recent observations in the new Millennium [10–12], entomological surveys performed in Guinea have shown the presence of the main malaria vectors, *Anopheles gambiae s.l*. and *An.funestus*, with an intense transmission potential [9]. As in many other sub-Saharan countries, several studies has been carried out in Guinea to assess the impact of malaria and explore possible strategies to interupt malaria transmission. Vezenegho et al. [12] evaluated malaria vector composition and insecticide susceptibility status in three localities in Guinea. Their aim was to provide data on malaria vector species composition and insecticide susceptibility status in Guinea. A similar study was conducted by Carnevale et al. [9] in order to estimate the diversity, infectivity rates and insecticide resistance levels in *Anopheles* species in Guinea. However, there is no recent study that reports the distribution of mosquito vectors in Conakry. Moreover, there is an increasing need for a thorough understanding of the ecological processes of malaria transmission in this urban area. Characterizing and mapping vector habitats will help to spatially rank malaria risk and focus control activities on a smaller scale [13]. To date no molecular identification of members of the *An*. *gambiae* complex had been undertaken in Conakry. Against this background, the current study aimed at establishing the relationship between breeding habitats, larval population size, species abundance, and seasonal variations in Conakry. It might also shed new light on the molecular forms and resistance status of the members of the *An*. *gambiae* s*.l*. complex and the infection rate of vectors. Insight gained will be useful in providing baseline data to support the national programme for malaria control in Conakry.

## Methods

### Ethical considerations

The study protocol was approved by the Ethics Committee of the Liverpool School of Tropical Medicine (1189RS). The study received ethics approval from the Ethics Committee of the Ministry of Health of Guinea (20/CNERS/12). Information about the study was delivered in the most spoken local languages: *Susu*, *Foula* and *Malinké*. Written informed consent was obtained from community leaders and heads of households before starting the study. Potential mosquito collectors were required to sign a consent form before working as collectors.

### Study sites

**Fig. 1:**
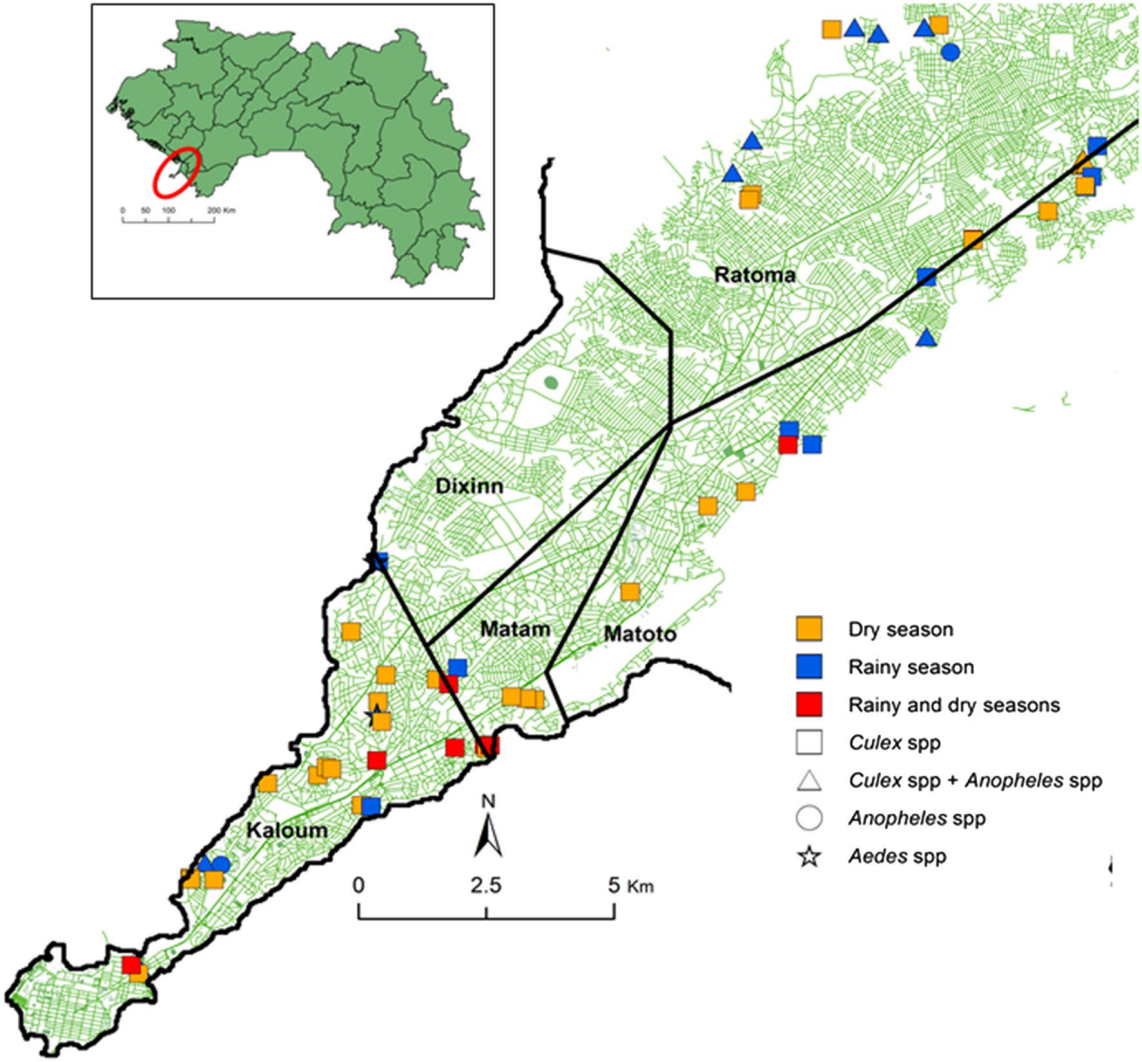
Distribution of mosquito breeding sites during the seasons in Conakry

The current study was carried out in Conakry, a peninsula of 308 km^2^ (Fig. 1), 34 km in length and 1–6 km wide. An estimated 2.5 million people live in Conakry, accounting for approximately one quarter of the total population of Guinea and 60 % of the urban population. Conakry is administratively divided into five districts: Dixinn, Kaloum, Matam, Matoto, and Ratoma. The district of Dixinn expands over 40.5 km^2^ with a population of 240,838, thus a density of 5946 inhabitants per km^2^. The district of Kaloum is 25 km^2^ with a population of 121,361 and a density of 4854 inhabitants per km^2^. The district of Matam has a surface of only 8 km^2^ with a population of 256,638 inhabitants, and hence, a very high density of 32,079 inhabitants per km^2^. The district of Matoto is 36 km^2^ with a population of 636,289 and a density of 17,674 inhabitants per km^2^. Ratoma is the largest district (62 km^2^) with a population of 531,279, hence a population density of 8569 inhabitants per km^2^. The average population density of Conakry is about 13,824 inhabitants per km^2^. The city, emerging from the continent, is surrounded at the end by the ocean and at the continental level by vast mangrove swamps. The city is crossed at both sides (west and east coasts) with stretches of the ocean that remain more or less humid throughout the year. Conakry is characterized by a hot and humid tropical climate, with a rainy season that lasts from May to November and a dry season from December to April [14]. ITNs constitute the main malaria preventive measure in Conakry, although the estimated coverage rate is only moderate (36 %) [6].

### Breeding site mapping

With the help of a detailed city map, a survey of larval breeding sites was undertaken. Larval breeding site prospecting was carried out for 6 days each in the dry season (January 2013) and in the rainy season (August 2013). The study area was inspected for open water bodies that were examined for mosquito larvae and pupae. A dipping technique for sampling larvae and pupae [15, 16], and visual observations of containers, were used for identification of breeding sites. The dipping technique was used for breeding sites that were densely populated by larvae and/or pupae, in order to identify the genera of mosquito larvae (e.g. *Culex*, *Anopheles*, and *Aedes*). In breeding sites that were large enough for the dipping technique to be employed, when larvae or pupae were not immediately found, 10 dips were performed, using a standard 200-ml dipper. The presence of larvae or pupae was defined by at least one larvae or pupae obtained in the 10 dips. In breeding sites where the dipping technique could not be used (e.g. containers), the water was transferred in a white clear tray for observation. All mosquito breeding sites were characterized according to their type, movement of water, turbidity, and exposure to sunlight, as described by Machault et al. [17]. Geographical coordinates of breeding sites were obtained by a hand-held global positioning system (GPS) receiver (Garmin GPS Map 60csx, Garmin International Inc., Olathe, USA) (Fig. 1).

### Adult mosquito collection

Based on the information gathered from the identification of larval breeding sites, 15 sectors were chosen according to potential exposure of the population to mosquito bites, and information provided by district leaders. From this information, mosquito collection sites were selected to represent different sectors of Conakry. This was to allow the collection of as many samples as possible.

Adult mosquitoes were collected monthly over a 1-year period from December 2012 to November 2013, using exit traps (ETC) and pyrethrum knock-down spray collections (PSC) [18]. Mosquitoes were collected monthly within 15 sites, selected in the five districts of Conakry. At each site, five traps were fixed on the windows of sleeping rooms, in five different households, for two consecutive days per district. Mosquitoes in the traps were collected every morning between 6 a.m. and 9 a.m. PSC were performed early each morning from 6 a.m. and 9. a.m. before opening the windows, in three rooms selected in different households during 2 days per district. ETCs were undertaken in different households than PSC,and the same households were used throughout the 1-year collections. However, in instances where individuals were absent or refused to participate, mosquitoes were collected in neighbouring households. The collected mosquitoes were identified at species level using readily available identification keys [19, 20]. After determining their feeding status, mosquitoes were dissected for parity.

### Species identification and determination of *kdr* status

Genomic DNA was extracted from the legs of the mosquitoes, morphologically identified as *An.gambiae*, using the boiling preparation method [21]. Briefly, the legs were crushed in 100 ml of distilled water and boiled at 95 °C for 10 min. The supernatant was used as template for the polymerase chain reaction-restriction fragment length polymorphism (PCR–RFLP) method. The extracted DNA was used for species identification using the PCR–RFLP method [22]. The determination of the knock-down mutation conferring resistance to pyrethroids was undertaken using the method of Martinez-Torres et al. [23].

### Determination of sporozoite rates

Individual *An.gambiae* mosquitoes were tested for *Plasmodium falciparum* and *P. vivax* sporozoites, using the VecTest™ dipstick assay (Medical Analysis Systems™, Camarillo, CA, USA), according to the manufacturer’s protocol. The Malaria Panel Assay is based on the dual monoclonal antibody sandwich principle [24]. For the confirmation of results, mosquito samples were further submitted to *Plasmodium* circumsporozoite enzyme-linked immunosorbent assay (ELISA), using the protocol described by Wirtz and colleagues [25].

### Malaria infection prevalence in Conakry

A systematic research of Ministry of Health Statistical Yearbooks was performed to determine malaria prevalence in children below the age of 5 years. The number of total consultations of children under the age of 5 years was compared to children examined with malaria over a 4-year period from 2009 to 2012 [26]. Furthermore, the malaria prevalence was compared to *Anopheles* densities at district level to appreciate the correlation between the two parameters.

### Statistical analysis

Data were entered into an Excel file and analysed using STATA version 13 (Stata Corp, College Station, TX, USA). A bivariate logistic regression model was used to identify parameters that determine the presence of *Culex* and *Anopheles* larvae and mosquito pupae. The Kruskal–Wallis equality of population rank test was used to compare adult mosquito densities between study sites and months. The Wilcoxon rank test was used to compare densities between seasons and the χ^2^ test for comparing parity rates in the dry and rainy season and the proportion of *Culex* and *Anopheles* breeding sites. ESRI ArcMap version 10.2.1 (Redlands, CA, USA) was employed for generating maps.

## Results

### Distribution of potential mosquito breeding sites in Conakry

Figure 1 shows the distribution of potential mosquito breeding sites in Conakry. Table 1 summarizes the numbers and percentage of sites where *Anopheles* and *Culex* larvae were found, stratified by habitat types and season. Overall, 94 potential breeding sites were examined; 53 (56.4 %) in the rainy season of which 3 (3.2 %) were only inhabited by *Aedes* and 41 (43.6 %) in the dry season. In the dry season, investigated sites were mainly composed of gutters (48.8 %), stretches of the ocean (19.5 %), pools of water (9.8 %), irrigated rice fields (7.3 %), water tanks (7.3 %), and septic tanks (7.3 %). In the rainy season, mosquito larvae and pupae were mainly found in blocked gutters (35.8 %), isolated pools of water (13.2 %), water tanks (13.2 %), irrigated rice fields (9.4 %), septic tanks (9.4 %), containers (7.6 %), and discarded tyres (5.7 %). In both seasons, there were significantly more breeding sites inhabited by *Culex* compared to *Anopheles* larvae (dry season: p < 0.001; rainy season: p = 0.001) (Table 1).

**Table 1:** Habitat characteristics and proportions of *Culex* and *Anopheles* larvae breeding sites in the dry and rainy seasons

Habitat characteristic	Dry season			Rainy season		
	Breeding site	*Culex*	*Anopheles*	Breeding site	*Culex*	*Anopheles*
Gutter	20 (48.8)	20 (100)	0 (0)	19 (38)	17 (89.5)	2 (10.5)
Stretch of ocean	8 (19.5)	8 (100)	0 (0)	3 (6)	3 (100)	0 (0)
Irrigated rice fields	3 (7.3)	3 (100)	0 (0)	5 (10)	4 (80)	5 (100)
Pools of water	4 (9.8)	4 (100)	3 (80)	7 (14)	4 (57.1)	3 (42.9)
Water tanks	3 (7.3)	3 (100)	0 (0)	7 (14)	7 (100)	2 (28.6)
Septic tanks	3 (7.3)	3 (100)	0 (0)	5 (9.4)	4 (80)	0 (0)
Tyres	0 (0)	0 (0)	0 (0)	3 (5.7)	2 (66.7)	0 (0)
Containers	0 (0)	0 (0)	0 (0)	4 (7.5)	2 (50)	1 (25)
Total	41	41 (100)	3 (7.3)	53	44 (83)	13 (24.5)

### Habitat characteristics of larval breeding sites

Table 2 shows the results of the bivariate logistic regression models used to identify factors that govern the presence of mosquito instars and the presence of pupae of *Anopheles* and *Culex*. It was found that *Culex* larvae develop in turbid waters [odds ratio (OR) 16.0; 95 % confidence interval (CI) 5.0–51.4], gutters (OR 6.8, 95 % CI 2.7–17.4) and in the dry season (OR 13.0; 95 % CI 1.6–104.1) as in the rainy season (OR 3.1; 95 % CI 1.7–5.8). These larvae were likely to develop in shady breeding sites (OR 4.8; 95 % CI 1.6–14.0). *Anopheles* larvae development is associated with irrigated rice fields (OR 30.8; 95 % CI 4.1–232.0) and pools of water (OR 15.4; 95 % CI 2.4–98.3). The development of *Anopheles* and *Culex* pupae is accelerated in turbid (OR 4.7; 95 % CI 2.3–9.6) and stagnant water (OR 13.5; 95 % CI 3.5–52.3) mainly during the dry season (OR 25.4; 95 % CI 5.6–116.4) (Table 2).

**Table 2:** Results of bivariate logistic regression models

Habitat characteristics	*Culex*			*Anopheles*			Pupae		
	OR^a^	95 % CI	*P* value	OR^a^	95 % CI	*P* value	OR^a^	95 % CI	*P* value
Season									
Dry	13	(1.6–104.1)	0.016	0.27	(0.07–1.03)	0.05	25.4	(5.6–116.4)	<0.001
Rainy	3.1	(1.7–5.8)	<0.001	0.29	(0.15–0.56)	<0.001	0.77	(0.44–0.32)	0.338
Turbidity									
Clear	0.18	(0.05–0.70)	0.014	1			0.19	(0.07–0.47)	<0.001
Turbid	16	(5–51.4)	<0.001	0.53	(0.29–1.00)	0.05	4.7	(2.3–9.6)	<0.001
Water movement									
Stagnant	12	(3.32–43.42)	<0.001	3.28	(0.39–26.93)	0.27	13.5	(3.5–52.3)	<0.001
Flowing	1	(0.37–2.66)	0.796	0.07	(0.01–0.50)	0.009	0.23	(0.06–0.81)	0.022
Sunlight									
Sunlight	1.28	(0.36–4.57)	0.699	1			1.04	(0.39–2.81)	0.931
Shaded	4.8	(1.6–14)	0.005	0.27	(0.15–0.47)	<0.001	1.87	(0.79–4.42)	0.151
Habitat									
Stretch of sea	0.39	(0.08–1.99)	0.259	1			0.92	(0.20–4.16)	0.913
Irrigated rice field	1.03	(0.10–10.23)	0.980	30.8	(4.1–232)	0.001	0.57	(0.12–2.85)	0.498
Pool of water	0.39	(0.08–1.99)	0.071	15.4	(2.4–98.3)	0.004	0.41	(0.10–1.66)	0.030
Water tank	1			4.62	(0.56–37.91)	0.154	0.52	(0.12–2.22)	0.375
Sceptic tank	1.03	(0.10–10.23)	0.980	1			1.03	(0.18–5.8)	0.970
Tyre	1			1			0.17	(0.01–2.11)	0.169
Container	0.44	(0.04–5.11)	0.513	6.17	(0.43–89.34)	0.182	1		
Gutter	6.8	(2.7–17.4)	<0.001	0.05	(0.01–0.22)	<0.001	2.9	(1.41–5.95)	0.004

*Outcome* presence vs absence of *Anopheles* larvae, *Culex* larvae and pupae (*Anopheles* and *Culex*); *explanatory variable* habitat characteristics

^a^ Crude odds ratio (OR)

^b^ *P* value based on likelihood ratio test (LRT)

### Adult mosquito fauna in Conakry

During the 1-year study, a total of 14,334 mosquitoes were collected by ETC (1524 nights) and 495 pyrethrum spray catches. ETC allowed the collection of 7594 (53.0 %) mosquitoes and PSC the remaining 6740 (47.0 %) mosquitoes. Ten species of mosquitoes belonging to four genera were identified: *Culex* (98.6 %), *Anopheles* (1.1 %), *Aedes* (0.19 %), and *Mansonia* (0.08 %). *Culex decens* (74.1 %) and *Culexquinquefasciatus* (24.3 %) were the two most common *Culex* species identified. *An.gambiaes.l.* was the only anopheline collected in the study area.

### Distribution of mosquitoes in Conakry

Mosquito densities varied from one site to another (Kruskal–Wallis (KW) test = 49.6, degree of freedom (df) = 14, p < 0.001) (Table 3). The highest density was obtained at Tombo [42.3 females/house/day (f/h/d)] in the district of Kaloum. The average density of mosquitoes collected in the districts of Dixinn, Kaloum, Matam, Ratoma and Matoto were 24.9, 24.3, 18.5, 17.4 and 16.2 f/h/d, respectively (p = 0.067).

**Table 3:** Distribution of *Culex spp*. and *Anopheles* density collected in the city of Conakry from December 2012 to November 2013

Site of collection	*Anopheles gambiae s.l.*			*Culex* spp.			Total of mosquitoes		
	ETC	PSC	Total (CI)	ETC	PSC	Total (CI)	ETC	PSC	Total (CI)
Matoto									
Tombolia	0	0	0 (0)	8.43	13.85	22.28 (13.18–31.39)	8.43	13.85	22.28 (13.18–31.39)
Gbessia	0	0	0 (0)	9.27	11.44	20.71 (7.99–32.43)	9.28	11.44	20.72 (8.01–32.44)
Bonagui	0	0	0 (0)	2.91	10.90	13.81 (7.53–20.1)	2.93	10.90	13.83 (7.56–20.11)
Lassanaya	0.07	0.03	0.10 (0–0.25)	3.19	5.49	8.68 (3.01–15.24)	3.27	5.51	8.78 (2.19–15.37)
Matam									
Boussoura	0.05	0	0.05 (0–0.13)	5.68	23.05	28.73 (10.35–47.13)	5.75	23.05	28.8 (10.37–47.24)
Bonfi	0	0	0 (0)	3.55	13.04	16.59 (2.18–31.55)	3.55	13.04	16.59 (2.18–28.80)
Mafanko	0	0	0 (0)	4.37	5.69	10.06 (3.5–16.62)	4.42	5.75	10.17 (3.62–16.72)
Ratoma									
Taouya	0.04	0	0.04 (0–0.09)	5.72	17.92	23.64 (12.43–34.82)	5.78	17.94	23.72 (12.53–34.91)
Dar es Alaam	0	0.04	0.04 (0–0.13)	4.87	12.68	17.55 (5.36–29.73)	4.87	12.72	17.59 (5.41–29.76)
Sonfonia I	0.79	1	1.79 (0.53–3.02)	2.67	10.875	13.55 (6.66–20.44)	3.59	11.90	15.49 (8.25–22.73)
Sonfonia II	0.21	0.21	0.42 (0.08–0.76)	3.69	8.71	12.40 (4.62–20.19)	3.91	8.92	12.83 (4.92–20.75)
Dixinn									
Belle vue	0.03	0	0.03 (0–0.06)	11.10	23.71	34.81 (11.38–58.25)	11.16	23.74	34.9 (11.5–58.28)
Camayenne	0	0	0 (0)	3.26	11.64	14.9 (9.98–19.83)	3.26	11.64	14.9 (9.98–19.83)
Kaloum									
Tombo	0.01	0.03	0.04 (0–0.12)	2.75	39.54	42.29 (16.73–67.85)	2.76	39.57	42.33 (16.79–67.86)
Coronthie	0.04	0	0.04 (0–0.09)	1.72	4.44	6.16 (2.77–9.54)	1.76	4.44	6.20 (2.82–9.58)

*Culex* densities ranged from 7.7 to 53.99 f/h/d in the collection sites. *Anopheles* mosquitoes were mainly obtained in Sonfonia I (1.8 f/h/d), Sonfonia II (0.4 f/h/d) and at Lassanayah barrage (0.1 f/h/d). In Tombolia, Gbessia, Bonagui, Bonfi, Mafanko, and Camayenne, no *Anopheles* mosquitoes were obtained.

### Variation of mosquito density

Mosquito density showed significant variation according to the month of collection (KW test = 43.85, df = 11, p < 0.001). The highest densities were obtained in the dry season months of December (30.4 f/h/d), January (45.1 f/h/d), February (32.8 f/h/d), March (24.3 f/h/d), and April (25.1 f/h/d). The average density in the dry season was statistically higher than the average density of mosquitoes collected in the rainy season (29.4 vs 12.0 f/h/d; Wilcoxon rank test = 2.84; p = 0.005). The lowest density (7.9 f/h/d) was collected in July, coinciding with the peak precipitation.

**Fig. 2:**
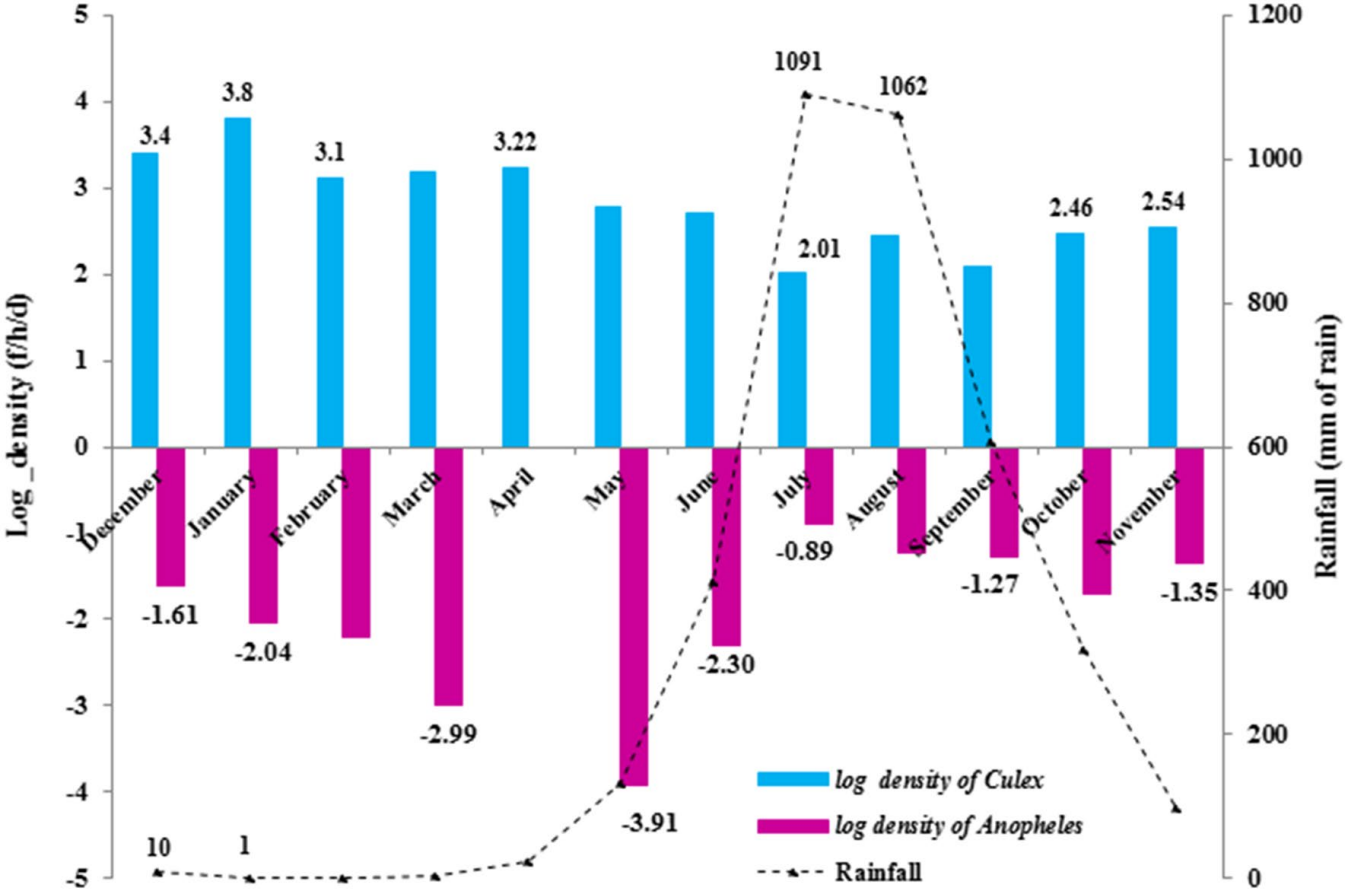
Variation of mosquito density according to the season

*Culex* mosquito density dominated that of *Anopheles* throughout the year. For *Culex* mosquitoes, the highest densities (ranging between 25.1 and 44.9 f/h/d) were obtained in the dry season. During the rainy season, the density decreased significantly to values ranging between 7.5 and 16.2 f/h/d. On the contrary, *An.gambiae* density was insignificant (0–0.2 f/h/d) in the dry season and increased slightly to 0.4 f/h/d in the rainy season. The densities of *Culex* and *Anopheles* were log transformed, which showed variability in abundance over both seasons (Fig. 2). Overall, there is a negative correlation between *Culex* density and rainfall (r = −0.67), while a positive correlation was observed for *Anopheles* (r = 0.76).

### Composition of feeding status and variation of *Culex* and *Anopheles* mosquitoes parity rate

Out of the 14,135 *Culex* collected, 39.2 % were unfed, 28.6 % were fed, 12.6 % were semi-gravid, and the remaining 19.6 % were gravid. For *An*. *gambiae*, out of 161 specimens, 33.5 % were unfed, 52.2 % were fed, 6.8 % were semi-gravid, and 7.5 % were gravid. The feeding rate of *An*. *gambiae* (59.3 %) was higher than that of *Culex* (56.0 %) (χ^2^ = 7.92; p = 0.005). For *Anopheles*, similar feeding rates were obtained by ETC and PSC (54.6 % vs 72.1 %; χ^2^ = 0.97; p = 0.324). For *Culex*, on the other hand, different feeding rates were observed using ETC and PSC (17.3 % vs 67.7 %; χ^2^ = 1600; p < 0.001).

**Fig. 3:**
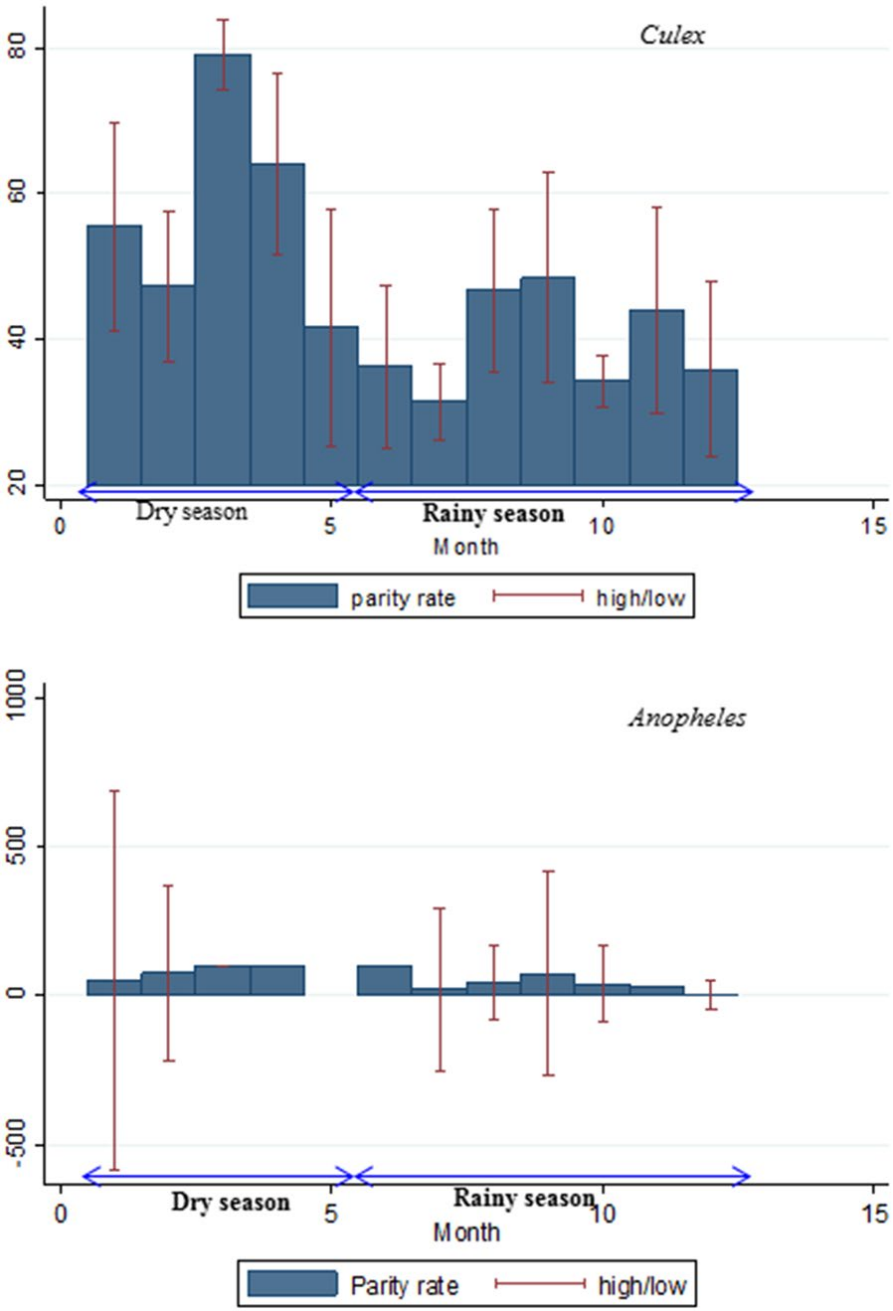
Evolution of *Culex* and *Anopheles* parity rate around the year

The results showed seasonal variation in the parity rate. For both species, the parity rate was higher during the dry season with 70.0 % of *An*. *gambiae* and 54.9 % of *Culex* spp. being parous. In the rainy season, significantly lower parity rates were observed for *Anopheles* (30.3 %; χ^2^ = 6.18; p < 0.013) and *Culex* mosquitoes (35.9 %; χ^2^ = 174.39; p < 0.001) (Fig. 3).

### Characterization of *Anopheles*

**Fig. 4:**
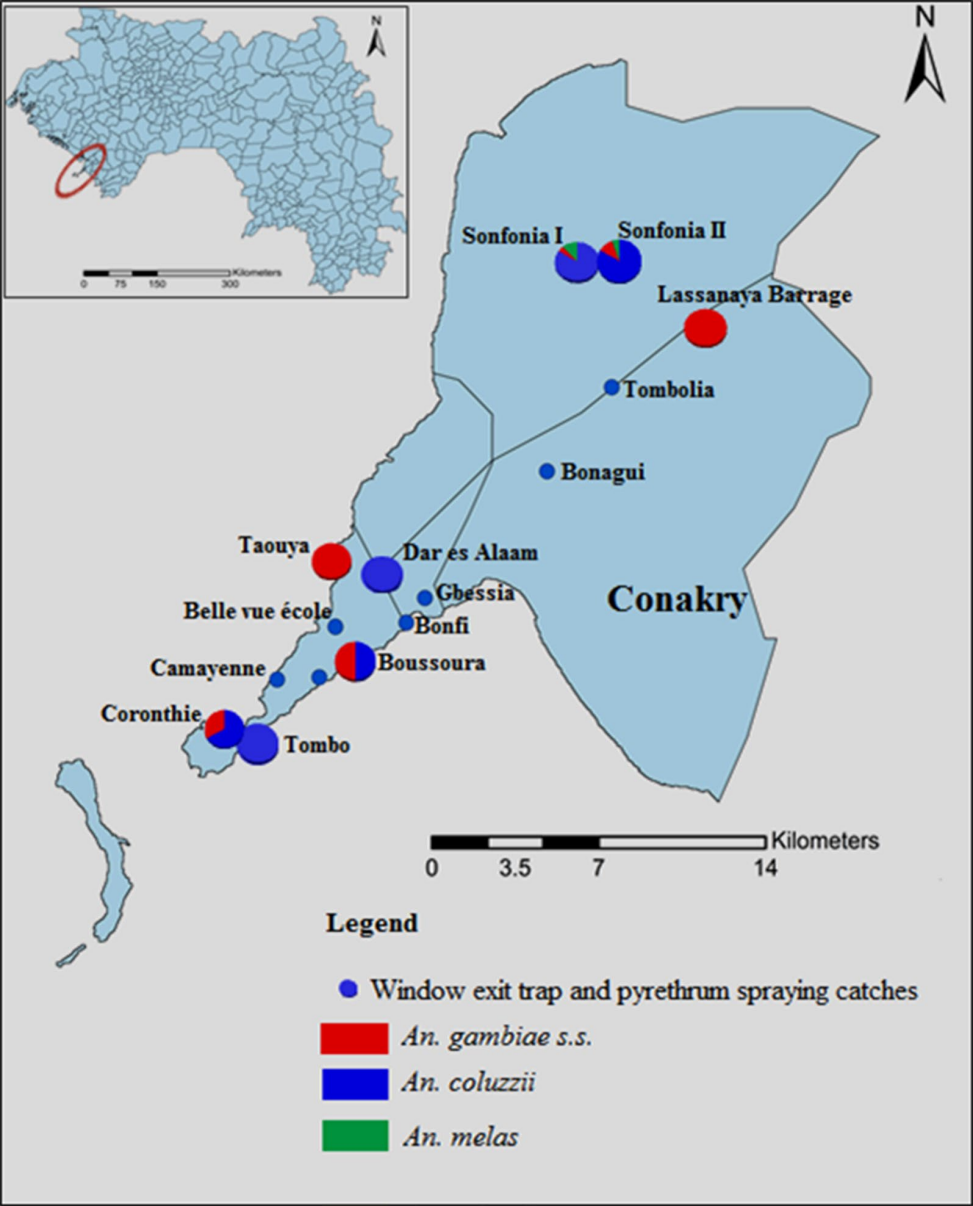
Distribution of the *Anopheles* *gambiae* species and molecular form in Conakry

Those 112 mosquitoes identified as *An*. *gambiaes.l.* were analysed for species identification. The overall frequency of the molecular M form (*An. coluzzii*; 79.5 %) was higher than that of the molecular S form (*An*. *gambiae s.s.*; 11.6 %) but the relative prevalence of these species varied throughout the collecting sites. Ten (8.9 %) were *An. melas* collected only at Sonfonia in the district of Ratoma (Fig. 4).

### Distribution of the kdr mutation

There were 102 mosquitoes identified as *An*. *coluzzii* and *An*. *gambiaes.s.,* and those were analysed for the Leu-Phe kdr mutation. The kdr mutation occurred in both species with respective frequencies of 65.2 and 81.8 %. The overall kdr mutation was 65.7 % (Table 4).

**Table 4:** Summary of the *kdr* genotypes for *Anopheles gambiae* molecular forms in Conakry

District	*An*. *gambiae* molecular form	Total	*Kdr* genotype			
			RR	RS	SS	(%) of resistant (R and RS)
Matoto	*An. gambiae s.s.*	4	4	0	0	4 (100)
Matam	*An. coluzzii*	1	0	0	1	1 (50)
	*An. gambiae s.s.*	1	0	0	1	0 (0)
Kaloum	*An. coluzzii*	1	0	0	1	1 (100)
	*An. gambiae s.s.*	4	2	0	0	2 (50)
Ratoma	*An. coluzzii*	84	28	28	25	56 (60.2)
	*An. gambiae s.s.*	7	2	2	1	4 (57.1)
Conakry	*An. coluzzii*	89	30	28	27	58 (65.2)
	*An. gambiae s.s.*	13	7	2	2	9 (81.8)
Total		102	37	30	29	67 (65.7)

### Determination of sporozoite rates

Prevalence of *P*. *falciparum* and *P.vivax* were assessed in all 112 *Anopheles* mosquitoes, using the VecTest™ kit and circumsporozoite ELISA. Both VecTest™ and ELISA analyses revealed that none of the *An.gambiae* specimens were infected, neither with *P.falciparum* nor with *P.vivax*.

### Malaria infection prevalence in Conakry

**Fig. 5:**
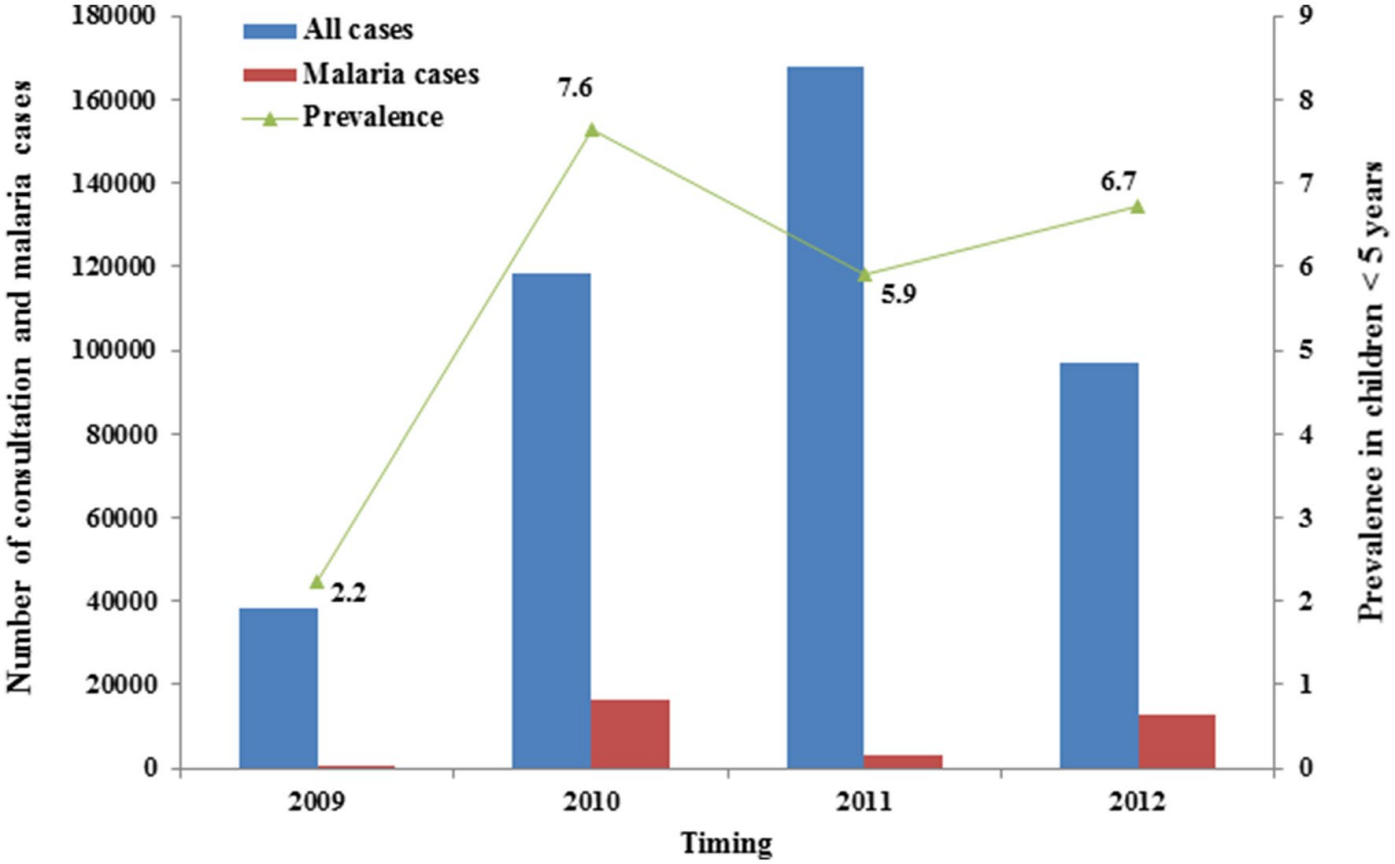
Malaria infection prevalence in children <5 years old, in Conakry

The malaria prevalence observed in children below the age of five years was relatively low: 2.2 % in 2009, 7.6 % in 2010, 5.9 % in 2011 and 6.7 % in 2012. The average prevalence over the 4-year observation period was 5.6 % (Fig. 5). The mean prevalence from 2009 to 2011 is 0 % in Kaloum, 3.8 % in Dixinn, 1.7 % in Matam and 7.8 % in Ratoma. There was a weak, negative correlation between district prevalence and *Anopheles* densities (r = −0.081).

## Discussion

### Bio-ecology of *Culex* and *Anopheles* mosquitoes

In the current study in Conakry, ETC and PSC revealed an abundance of mosquitoes, dominated by *Culex*, particularly *Cx.decens* and *Cx*. *quinquefasciatus*. On the other hand, *An.gambiae*, the main malaria vector, was caught only rarely.

The distribution pattern of adult mosquitoes is related to habitat preferences of immature stages. Habitats may be natural or man-made, temporary or permanent. Moreover, each species has specific habitat preferences [27]. Depending on the availability of breeding sites, *Culex* and *Anopheles* show distinct spatial heterogeities. *Culex* were found in most of the collection sites, which might be explained by the extent of polluted breeding sources that is common for large cities in Africa. Adult *An.gambiae* were collected mainly in Sonfonia and Lassanaya, both located in the periphery of Conakry. Urban growth in developing countries often results in development and sprawl of poor quality housing, inadequate water supplies and sanitation, and overcrowding [28]. In such areas, human activities are quite similar to those observed in traditional villages, characterized by the presence of small garden plots, irrigation trenches, and excavations [29], and continue to provide breeding sites for malaria vectors [30]. Both collection sites are located near irrigated agricultural spaces, where *Anopheles* breeding sites were observed. With regard to breeding requirements, there is evidence of adaptation of *Anopheles* species to urban settings [31]. Malaria vector density is typically higher in these peri-urban areas where malaria transmission remains a significant problem [32]. The importance of urban agricultural activity on malaria transmission has been reported before in cities of Burkina Faso, Côte d’Ivoire and Ghana [33–35].

In the present study, mosquito densities varied according to the month (and season) of collection with a negative correlation between the amount of precipitation and average density of adult mosquitoes. The highest densities were observed during the dry season. During the rainy season, the average density of mosquitoes was about half that observed in the dry season. A similar observation has been made in Bangladesh in a study addressing the seasonal abundance of mosquitoes and their association with meteorological features [36]. The lower adult mosquito density may be attributed to rainfall washing away mosquito eggs and larvae from breeding sites [37], mainly for *Culex* mosquitoes. Indeed, in all collection sites, *Culex* density in the rainy season was significantly lower, while *Anopheles* density showed a relative increase, mainly in areas in close proximity to irrigated rice fields. The higher number of *Anopheles* breeding sites in the rainy season compared to the dry season is in accordance with previous observations made in western Côte d’Ivoire [15] and in southern Sri Lanka [38]. Rains are known to have a dual effect on the development of mosquito larvae. New mosquito breeding sites are created during periods of rain, while previously existing sites are washed away [37]. It has been observed that newly formed breeding sites were rapidly colonized by *Anopheles* larvae (as these mosquitoes prefer such habitats). In the rainy season, *Anopheles* larvae were found not only in irrigated areas, but also in ditches, blocked gutters and stable, clear pools of water (most of them temporary), highlighting the importance of temporary water collections in the breeding of *Anopheles* mosquitoes [39]. In most tropical areas, mosquito populations are expected to oscillate cyclically as precipitation fluctuates, because the number of available breeding sites is a function of rainfall [40].

### Characterization of *Anopheles* in Conakry

The data presented here show that both *An*. *coluzzii* (M molecular form) and *An.gambiae s.s*. (S molecular forms) are present in Conakry, confirming previous observations from studies conducted elsewhere in West Africa [12, 41, 42]. Interestingly, *An*. *coluzzii* was more prevalent in Conakry compared to *An.gambiae s.s*., which is consistent with findings of de Souza et al., who reported that *An*. *coluzzii* dominated *An*. *gambiaes.s*. in Monrovia, Liberia [21]. The relative dominance of one species over another is believed to be associated with breeding site characteristics [43]. In general, *An.gambiae s.s.* is not well adapted to rice paddies in West Africa, whilst *An.coluzzii* develops well [44]. In a study addressing *Anopheles* breeding pattern, Gimonneau et al. [45] demonstrated that the rice field appeared to be the core habitat of *An.coluzzii* from which it successfully emerged and spread in the surrounding area where *An.gambiae s.s.* was mostly thriving. The current findings confirm this observation, since the majority of *An.coluzzii* collected in Conakry were from rice-growing areas nearby Sonfonia. Diabate et al. also reported that *An.coluzzii* mosquitoes tend to be associated with flooded or irrigated sites that provide permanent breeding conditions, whereas *An.gambiae s.s*. mosquitoes are associated with rain-dependent temporary sites [42]. Additionally, *An. melas* mosquitoes were observed, which confirms previous observations that *An*. *melas* primarily occurs in coastal areas bordering the ocean that are subject to flooding [46].

In this study, *kdr* mutations were found in both *An.coluzzii* and *An.gambiae s.s.* The kdr mutation was initially thought to be present only in the *An*. *gambiae* molecular S form. However, studies have shown its occurrence in the M molecular form [47]. It is assumed that the presence of the mutation in the M form may be a result of introgression from the S form [48]. However, observations from the Island of Bioko, Equatorial Guinea, where the mutation was observed only in the M form [49] and the absence of the *kdr* gene in the nearest mainland population in Tiko, Cameroon, suggests that the emergence of *kdr* resistance in the M population of *An*. *gambiae* occurred as an independent evolutionary event. It must be noted that rice fields around Sonfonia, where most of the *Anopheles* mosquitoes were collected, is flooded mangrove swamp where neither chemical fertilizers, pesticides, nor herbicides were used [50]. These levels of *kdr* resistance to pyrethroids and DDT might be due to the usage of ITNs that has been progressively scaled up in the study area [51]. Further investigations need to address this issue in greater depth. Other studies have shown a strong increase in the frequency of the *kdr* gene immediately following the implementation of an ITN campaign [52]. Although these results may represent initial information, it was recently demonstrated that there is no significant association between the presence of the 1014F *kdr* allele and ability to survive exposure to pyrethroid [47]. Insecticide susceptibility testing and bio-assay data are necessary to validate these findings. Of note, insecticide susceptibility testing requires a collection of a sufficiently large number of *Anopheles* larvae and additional laboratory equipment, which were not available when the current study was conducted. Emerging individuals from resting, fed-female mosquitoes could also help in addressing this issue.

In characterizing the malaria vector species in Conakry, *An.gambiae* mosquitoes were tested for *P.falciparum* and *P*. *vivax* sporozoite infection. None of the mosquitoes tested was found positive. The absence of infection may be associated with the very low numbers of *Anopheles* collected. In the present study, *An*. *gambiae* and *Culex* spp. showed significant variation in their parity rate, which was higher during the dry season compared to the rainy season. The parity rate of *An.gambiae and Culex* spp. thus suggests higher longevity during the dry season, likely to maintain mosquito-borne disease transmission during this season [53]. However, most of the *Anopheles* mosquitoes were collected in the rainy season, while the population average longevity was significantly low, thus reducing the odds of finding mosquitoes with sporozoite infection. Mosquito collection employing other methods, such as human landing catches, could help determine the vector infection and infectivity rates. Malaria is nonetheless present in Conakry, with an average prevalence of 5.6 % in children aged <5 years as reported by the national malaria control programme over a 4-year period (2009–2012) [54], holding the lowest prevalence of malaria in Guinea. A similar prevalence rate has been reported by Carnevale et al. [9]. This low prevalence of malaria might support the results of this study, indicating relatively low density of *Anopheles* vector mosquitoes in Conakry. It is, however, difficult to confirm with the results presented here whether there is local transmission or whether malaria cases in Conakry are imported from rural areas. For example, while there is high prevalence of malaria in Dixinn, it must be noted that this district is central in Conakry, where the main hospital and the department of infectious diseases of the city are concentrated. New research is required to address the feasibility of malaria elimination in Conakry. Through sustained control measures, many islands are kept malaria-free, despite the presence of competent vector species [55]. The low malaria prevalences associated with low *Anopheles* vector density present prospects for malaria elimination in Conakry.

## Conclusions

Conakry is marked by an abundance of *Culex* mosquitoes. The distribution pattern of adult mosquitoes is related to habitat preferences of the immature stages. The dry season would be critical for mosquito-borne diseases regarding the longevity and abundance of these mosquitoes. The findings of the present study underscore the importance of irrigation schemes in urban areas. The distribution of *An.gambiae s.s*., *An*. *melas* prevalence and the kdr gene are reported for the first time in Conakry. A high prevalence of kdr mutation has been observed in the study site, but there is a limitation to these results because the determination of the kdr frequencies was not undertaken on surviving or dead mosquitoes exposed to pyrethroids through insecticide susceptibility testing. *Anopheles* density was very low over the year and relatively higher in the rainy season where mosquitoes parity rate was low. None of the tested mosquitoes were infected with sporozoïtes. These findings, in concert with the national malaria report, indicate low malaria prevalence over the years and offer new prospects for malaria elimination in Conakry.
